# tVNS alters inflammatory response in adult VPA‐induced mouse model of autism: evidence for sexual dimorphism

**DOI:** 10.1002/2211-5463.13889

**Published:** 2024-10-14

**Authors:** Hale Gök Dağıdır, Neslihan Bukan, Meltem Bahcelioglu, Ayşen Çalıkuşu, Ece Alim, Saadet Özen Dizakar, Elif Topa, Hayrunnisa Bolay

**Affiliations:** ^1^ Department of Medical Biochemistry, Faculty of Medicine Gazi University Ankara Turkey; ^2^ Neuroscience and Neurotechnology Center of Excellence (NÖROM) Gazi University Ankara Turkey; ^3^ Faculty of Medicine, Department of Anatomy, and Neuroscience and Neurotechnology Center of Excellence NÖROM Gazi University Ankara Turkey; ^4^ Department of Neuroscience, Institute of Health Sciences Gazi University Ankara Turkey; ^5^ Department of Histology and Embryology, Faculty of Medicine İzmir Bakırcay University Turkey; ^6^ Neuropsychiatry Education, Research and Application Center (NPM) Gazi University Ankara Turkey; ^7^ Department of Neurology and Algology, Neuropsychiatry Education, Research and Application Center (NPM), Neuroscience and Neurotechnology Center of Excellence NÖROM Gazi University Ankara Turkey

**Keywords:** autism, brain NLRP3, cytokines, inflammation, transauricular vagal nerve stimulation, VPA

## Abstract

Autism is a neurodevelopmental disorder with limited treatment alternatives and which incidence is increasing. Some research suggests that vagus nerve simulation might lead to the reduction of certain symptom. Therefore, we aimed to examine the effect of bilateral transcutaneous auricular vagus nerve stimulation (tVNS) on the inflammatory response in an adult valproic acid (VPA) induced mouse (C57BL6) model of autism for the first time. The autism model was induced by oral VPA administration (600 mg·kg^−1^) to C57BL/6 pregnant mice on E12.5 days. The study included three groups: the VPA Transcutaneous Auricular Stimulation Group (VPA + tVNS), the VPA Control Group (VPA + sham), and the Healthy Control Group (Control + sham). Each group included 16 mice (8 M/8 F). Our results show that serum IL‐1β and IL‐6 levels were significantly higher in male VPA‐exposed mice than controls. However, IL‐1β was significantly lower, and IL‐6, TNF‐ α, and IL‐22 were not different in female VPA‐exposed mice compared to the control group. Brain NLRP3 levels were significantly higher in both sexes in the VPA autism model (*P* < 0.05). tVNS application increased brain NLRP3 levels in both sexes and reduced serum IL‐1β levels in male mice. We conclude that cytokine dysregulation is associated with the VPA‐induced adult autism model, and the inflammatory response is more pronounced in male mice. tVNS application altered the inflammatory response and increased brain NLPR3 levels in both sexes. Further studies are needed to understand the beneficial or detrimental role of the inflammatory response in autism and its sexual dimorphism.

## Graphical abstract

Cytokine levels in adult valproic acid (VPA)‐induced mouse (C57BL6) model of autism has been investigated and found to be altered. We show that bilateral transcutaneous auricular vagus nerve stimulation (tVNS) altered the inflammatory response and increased brain NLPR3 levels.

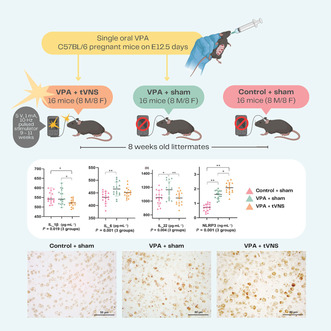

**Abbreviations**
ASD: autism spectrum disorder
ELISA: enzyme‐linked immuno sorbent assay
IHC: immunohistochemistry
IL: interleukin
IP: intraperitoneal
NLRP3: NOD‐, LRR‐ and pyrin domain‐containing protein 3
PND: postnatal day
TNF‐α: tumor necrosis factor alpha
tVNS: transauricular vagal nerve stimulation
VN: Vagus nerve
VPA: valproic acid

Autism is a very diverse neurodevelopmental disorder. It was first defined by psychiatrist Leo Kanner in 1943, and its prevalence has steadily increased in recent years [1, 2, 3, 4]. Medications can be used for comorbid conditions, but there is no single medical treatment which is effective for all symptoms of Autism Spectrum Disorder (ASD) [2, 5]. Screening and monitoring are crucial components for early detection of developmental disorders in children [6].

Because of the high variability in behavior, biological findings, and response to treatment, many experts postulate many different theories of autism, each with a slightly different etiology [7, 8]. Among the underlying causes of autism, interactions between genetic predisposition, epigenetic, cellular stress, neurological, metabolic, psychosocial, prenatal/postnatal, environmental, and immunological factors are shown [2, 9, 10, 11, 12, 13]. It is thought that one or more factors may cause autism through mutual interaction; all these interactions are thought to cause an unbalanced neurotransmitter profile, loss of function in neuronal pathways, and defective neuronal connections [14].

The pathogenic mechanism of autism has not yet been elucidated, and there is no specific treatment yet. Biomarkers are needed for early diagnosis [12]. Therefore, genetic, neural, pharmacological manipulations, and animal studies are necessary for ASD research.

Valproic acid is a well‐known drug triggering of neural tube defects, and its exposure has also been associated with autism‐like behaviors [15]. Therefore, some studies use valproic acid (VPA) during pregnancy or the postpartum period as exposure to VPA at different stages of brain development triggers autism‐like behaviors [16, 17, 18, 19]. Embryogenesis is predicted to be a critical period for autism, so creating autism‐like symptoms using VPA is a promising neurotoxicological model to evaluate the mechanisms that play a role in the development of autism [17, 18]. Other commonly employed models in autism research include genetic monogenic models, chromosomal diseases, neurotoxicological models using propionic acid, viral models, fecal microbiota transplantation, and LPS and idiopathic models using inbred strains [20, 21, 22, 23].

Although treatment for core features have not been found for ASD, there are interventional studies that lead to a positive reduction in symptoms; ‘Vagal stimulation’ (VNS) is one of these [24]. The vagus, the 10th cranial nerve, establishes bidirectional communication between the viscera and the brain; and is an essential part of the autonomic nervous system [25]. The relationship between vagus and autism is tried to be explained by two theories: polyvagal theory and neurovisceral integration model [26]. According to polyvagal theory, individual differences in social participation skills can be explained through the vagal nerve [27]. This theory suggests that the vagal nerve has evolved to allow parasympathetic signals to travel to the heart more efficiently, thus promoting adaptive social behavior [25, 28]. The neurovisceral model focuses on the relationship between cognition, emotion regulation, and behavior; and the parasympathetic nervous system (PNS) is an essential part of this network [29]. Individuals with autism usually have an irregular sympathetic and parasympathetic system, such as irregular heart rate, dysrhythmic respiratory, or vagal tone [27, 30, 31, 32]. Electrocardiogram data, plethysmograph, and cardiac vagal tone measurements obtained from individuals with autism also support this view [27, 32, 33]. PNS is associated with emotion, and the vagus nerve, which is the main nerve of PNS, transmits the interoceptive signals from the central autonomic network [26, 34]. According to some research, decreased vagal activity is associated with language disorders and autistic behaviors [27, 35, 36, 37]. VNS is a neuromodulation technique that stimulates autonomic pathways through an electrode around the vagus nerve [26]. Researchers are showing that VNS may provide some symptomatic relief in individuals with autism [35, 38, 39]. In a pilot study conducted with children with ASD, tVNS application improved anxiety and sleep scores [40]. In a separate study involving children with autism, it was found that VNS reduced seizure frequency and enhanced quality of life [41]. In a mouse model of inflammation induced by LPS, the administration of pVNS resulted in changes in microglial morphology [42].

Available data point to a possible relationship between cytokine changes and autism. However, systematic investigations of neuroimmunological factors are needed [43]. In recent years, new evidence has been found on inflammatory mechanisms that contribute to ASD [44]. Changes in the immune system, elevated pro‐inflammatory cytokine levels, epidemiological evidence of increased immune‐related problems in mothers of children with ASD, postmortem observations of neuroinflammatory states, and altered cytokine levels in the brains all show the relationships between ASD and immunity [44, 45, 46]. Numerous cytokines, chemokines, and inflammation studies show a correlation between cytokine levels and ASD status. Thus, cytokines have significant potential as biomarkers in the field [47, 48, 49, 50].

The efficacy of VNS has been shown in the quality of life and cognition, but some studies have found contradictory results [38, 51, 52]. For instance, in a histopathological study conducted with autopsies of chronic epilepsy cases receiving long‐term VNS treatment, no differences were observed in the brainstem nuclei compared to controls [53]. Studies draw attention to immune dysregulation in autism, but there is a need for new studies that provide evidence for this process and compare genders [54, 55, 56]. Considering all these, this research aims to obtain data about neuroinflammation and VNS in a VPA‐induced autism animal model and to contribute to the literature about its symptoms and treatment by trying to provide information about the pathophysiology of autism while examining pro‐inflammatory and anti‐inflammatory cytokines.

## Materials and methods

### Animals

C57BL/6 mice were used in the experiments. Mice were kept on a 12/12 h light–dark cycle and had free access to food and water. Mice underwent tVNS intervention, and then, brain tissues and serum samples were collected to measure IL‐1β, IL‐6, IL‐22, TNF‐α, and brain NLRP3 levels. The ethical approval was obtained from the Gazi University Local Ethics Committee for Animal Experiments (Number: G.Ü.E.T‐21.031).

### Drugs

The first gestational day (E0) was recorded. On embryonic day 12.5 (E12.5), pregnant mice were orally administered 600 mg·kg^−1^ VPA (Depakine 500 mg, SANOFI, Paris, France) or saline.

### Offspring

After birth (PND1), offspring were labeled separately by writing numbers on their tails, their sexes (according to anogenital distance) were determined, and their body weights were recorded. All offspring mice were left with their mothers until weaning at PND28; no treatment was done. On PND28, weaned offspring were placed separately, at least four offspring in the same cage (4 same‐sex mice per 1 cage).

### Experimental design

**Fig. 1:**
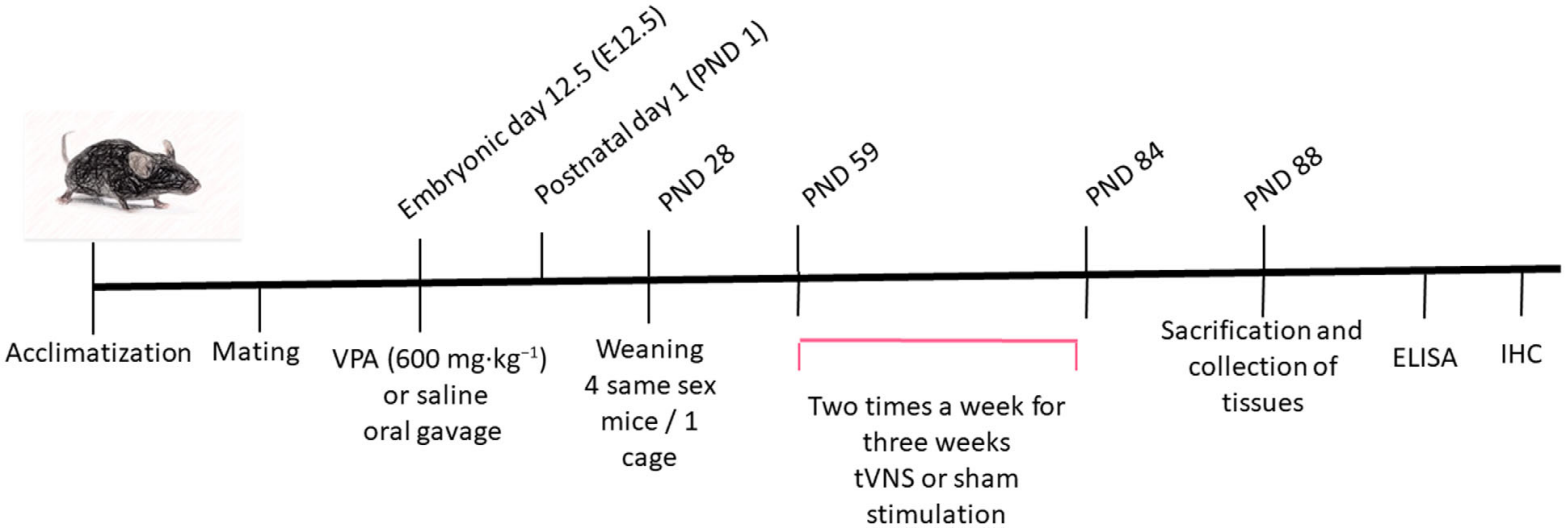
Timeline of experimental design.

Offspring born to pregnant mice given VPA were randomly assigned to VPA + tVNS and VPA + sham group. Pups born to pregnant mice who were given saline were included in the Control + sham group. Between the 9th and 11th weeks, tVNS was administered in the VPA + tVNS group. In the 12th week, mice were sacrificed, and tissues were collected for ELISA and immunohistochemical analysis. The three groups formed with eight females and eight males in each group are as follows (*N* = 48) (Fig. 1).

VPA + tVNS group: Pups born to mice given VPA underwent tVNS (*n* = 16, 8 M/8 F).

VPA + sham group: Pups born to mice given VPA underwent sham stimulation (*n* = 16, 8 M/8F).

Control + sham group: Pups born to mice given saline underwent sham stimulation (*n* = 16, 8 M/8 F).

### tVNS

The study performed tVNS under general anesthesia (Pfizer Ketalar 500 mg injectable vial). The anesthetized mice were placed on cardboard, and then, electrodes coated with gel were placed on the concha of both ears of the mice. Stimulation was performed with a 5 V, 1 mA, 10 Hz pulsed stimulator (Vagustim, Istanbul, Turkey). The tVNS was performed twice weekly for 3 weeks from PND 59 to PND 84 (9–11 weeks). In the sham stimulation groups, electrode placements were made, but no stimulation was performed. After stimulation, the mice were observed to come out of anesthesia and were returned to their cages after waking up.

### ELISA

Mice were anesthetized by intraperitoneal (ip) ketamine (Pfizer, Ketalar 500 mg injectable vial), cardiac blood was taken, and brain tissues were harvested. Blood was kept at room temperature for 2 h and then centrifuged (1000 ***g*** 20 min). Brain and serum samples were stored at −80 °C (88 400 V‐86, THERMO, Asheville, NC, USA) until ELISA (Enzyme‐Linked Immuno Sorbent Assay) was analyzed. For each mouse, randomly selected hemispheres were reserved for ELISA analysis, and the other hemisphere was reserved for immunohistochemistry.

Brain tissues (randomly selected hemisphere) were homogenized in Tissue Protein Extraction Reagent (T‐PER TM, w:v = 1/20) on ice before analysis. Protease Inhibitor Cocktail EDTA Free was added (Thermo Scientific Halt TM 78437, 10 μL per 1 mL of lysis buffer) and then centrifuged (10 000 ***g*** for 5 min).

Serum IL‐1β (SEA563MU), IL‐6 (SEA079MU), IL‐22 (SEC032MU), TNF‐α (SEA133MU) levels, and brain NLRP3 (SEK115MU) levels were measured by high‐sensitivity sandwich‐type ELISA Mouse Kits (Cloud Clone Corb, Houston, TX, USA). Standards and samples were studied in duplicate. All reagents were brought to room temperature before the analysis. Standards and samples were added to micro ELISA plate wells and incubated with specific antibodies. The order of addition of the solutions, the number of washes, and the incubation times were advanced according to the assay procedure of each kit. After the last wash, substrate solution was added to each well, and a blue color reaction was obtained. Finally, a stop solution was added, and the optical density (OD) was measured using a microplate reader set at 450 nm. A Combiwash (Human Diagnostics Worldwide, Wiesbaden, Germany) ELISA plate washer and Chromate (Awareness Technology, Inc., Palm City, Florida, USA) reader were used during the analysis.

### Immunohistochemistry (IHC)

PFA‐fixed brains were processed for embedding in paraffin blocks. Brains were cut into 4 μm coronal sections on slides. All the slides were deparaffinized, rehydrated, and microwaved in citrate buffer (pH 6.0) for 10 min to perform antigen retrieval. The slides were treated with 3% hydrogen peroxide, followed by a blocking solution (TP‐125‐UB, Thermo Scientific) and incubated with rabbit anti‐NLRP3 (1 : 1000, bs‐10021R, Bioss, China) primary antibody overnight at 4 °C. Afterwards, a secondary antibody (anti‐rabbit IgG (TP‐125‐BN, Thermo Scientific)) was applied. After rinsing with phosphate‐buffered saline (PBS), the streptavidin peroxidase complex revealed the reaction product. The samples were incubated with diaminobenzidine tetrahydrochloride (DAB) chromogen. Stained specimens were observed under a light microscope (Axio Scope.A1, Zeiss, Oberkochen, Germany) and photographed using a software (Zen 2.6 (blue edition) software, Germany).

### Statistical analysis of research data

A statistical analysis of the research was performed using the spss statistic software 25.0 (IBM, Armonk, Westchester, New York, USA) package program. Significance was assumed for all values where *P* < 0.05.

The Kolmogorov–Smirnov test examined whether the results were by normal distribution, and histogram distribution graphs and non‐parametric statistical methods were used for cases that did not show normal distribution. Continuous variables are expressed as median and interquartile range (IQR) or mean ± SD according to their distribution structure. The Kruskal–Wallis test determined the significance of differences between groups with non‐parametric distribution. Mann–Whitney *U* test was used to examine differences between two independent groups. The analysis of parametric variables was performed using One‐way ANOVA. Bonferroni test was used in *post‐hoc* analysis.

## Results

**Fig. 2:**
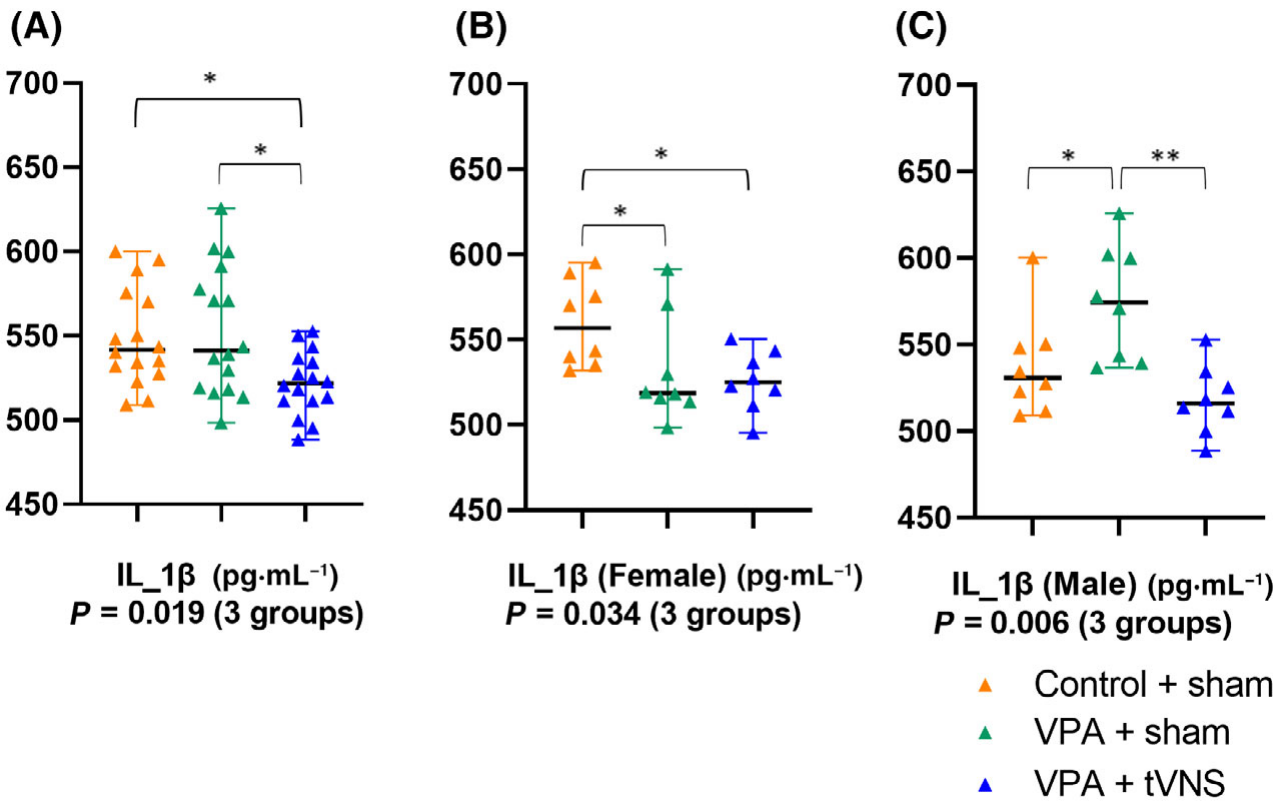
(A) Serum IL‐1β levels were significantly different between Control + sham (*n* = 16), VPA + sham (*n* = 16), VPA + tVNS (*n* = 16) (*P* = 0.019). There was a significant difference between groups in (B) female mice (*P* = 0.034) and (C) male mice (*P* = 0.006). Statistical analysis was performed with the Kruskal–Wallis test (3 groups). Groups were compared in pairs with the Mann–Whitney *U* test. Data were shown as median (Q1–Q3). *P‐value < 0.05, **P < 0.01.

**Fig. 3:**
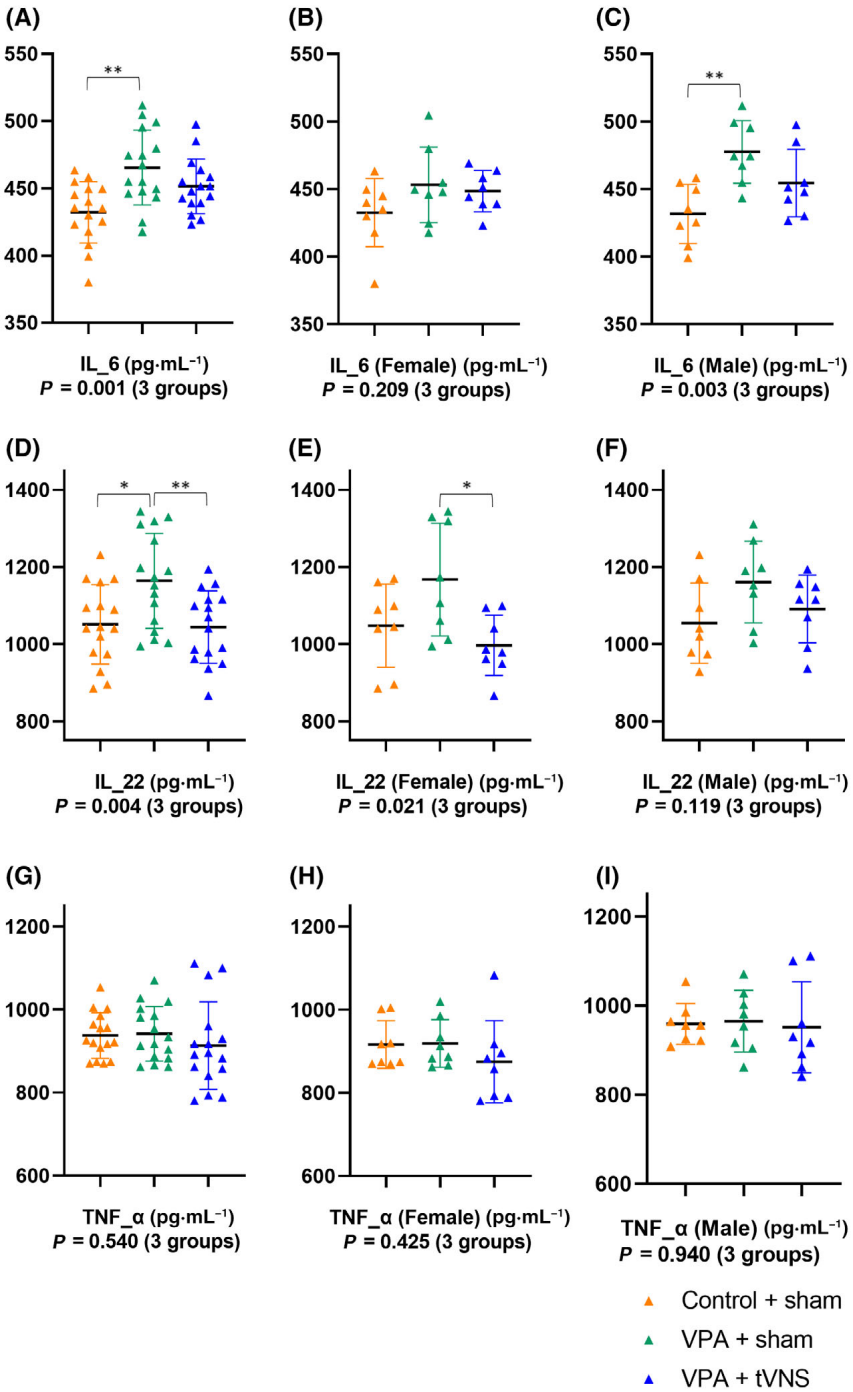
(A) Serum IL‐6 levels (*F* = 7.841, *P* = 0.001) were significantly different between Control + sham (*n* = 16), VPA + sham (*n* = 16), VPA + tVNS (*n* = 16). There was comparable in (B) female mice (*P* = 0.209) but significantly different in (C) male mice (*P* = 0.003) between groups. (D) Serum IL‐22 levels (*F* = 6.321, *P* = 0.004) were significantly different between groups. There was significantly different in (E) female mice (*P* = 0.021) but comparable in (F) male mice (*P* = 0.119) between groups. (G) Serum TNF‐α levels (*F* = 0.624, *P* = 0.540) were similar between the three groups. There was comparable in (H) female mice (*P* = 0.425) and (I) male mice (*P* = 0.940) between groups. Statistical analysis was performed with One‐way ANOVA. *Post‐hoc* analyses were performed using the Bonferroni test. Data were shown as mean ± SD. *P‐value < 0.05, **P < 0.01.

Serum IL‐1β (*P* = 0.019), IL‐6 (*P* = 0.001), IL‐22 (*P* = 0.004) levels and brain NLRP3 (*P* = 0.001) levels were significantly different across groups (*P* < 0.05) (Figs 2 and 3).

We demonstrated the different effects of gender on cytokines in IL‐1β levels in the VPA group compared to the Control + sham. Serum IL‐1β levels were significantly decreased in VPA + sham group female mice compared to Control + sham (median 518.68 (514.25–560.55) vs. 556.70 (536.25–585.70), *P* = 0.038), but were significantly increased in VPA + sham group male mice compared to Control + sham (median 574.37 (540.18–601.28) vs. 530.83 (514.25–549.69), *P* = 0.036), groups were compared in pairs with Mann–Whitney *U* test and data were shown as median (Q1–Q3).

Serum IL‐6 (465.40 ± 27.89 vs. 432.18 ± 22.76, *P* = 0.001) and IL‐22 (1164.43 ± 123.38 vs. 1051.41 ± 102.61, *P* = 0.014) levels were significantly higher in the VPA + sham group compared to the Control + sham group, *post‐hoc* analysis were performed using Bonferroni multiple comparisons test and data were shown as mean ± SD.

There was no significant difference between the three groups regarding serum TNF‐α levels (*P* = 0.540); statistical analysis was performed with One‐way ANOVA. But when male and female mice in all groups were compared, TNF‐α levels were significantly higher in males (*P* = 0.012). Additionally, NLRP3 (*P* = 0.001) levels were significantly different when females and males were compared in the Control + Sham group.

**Fig. 4:**
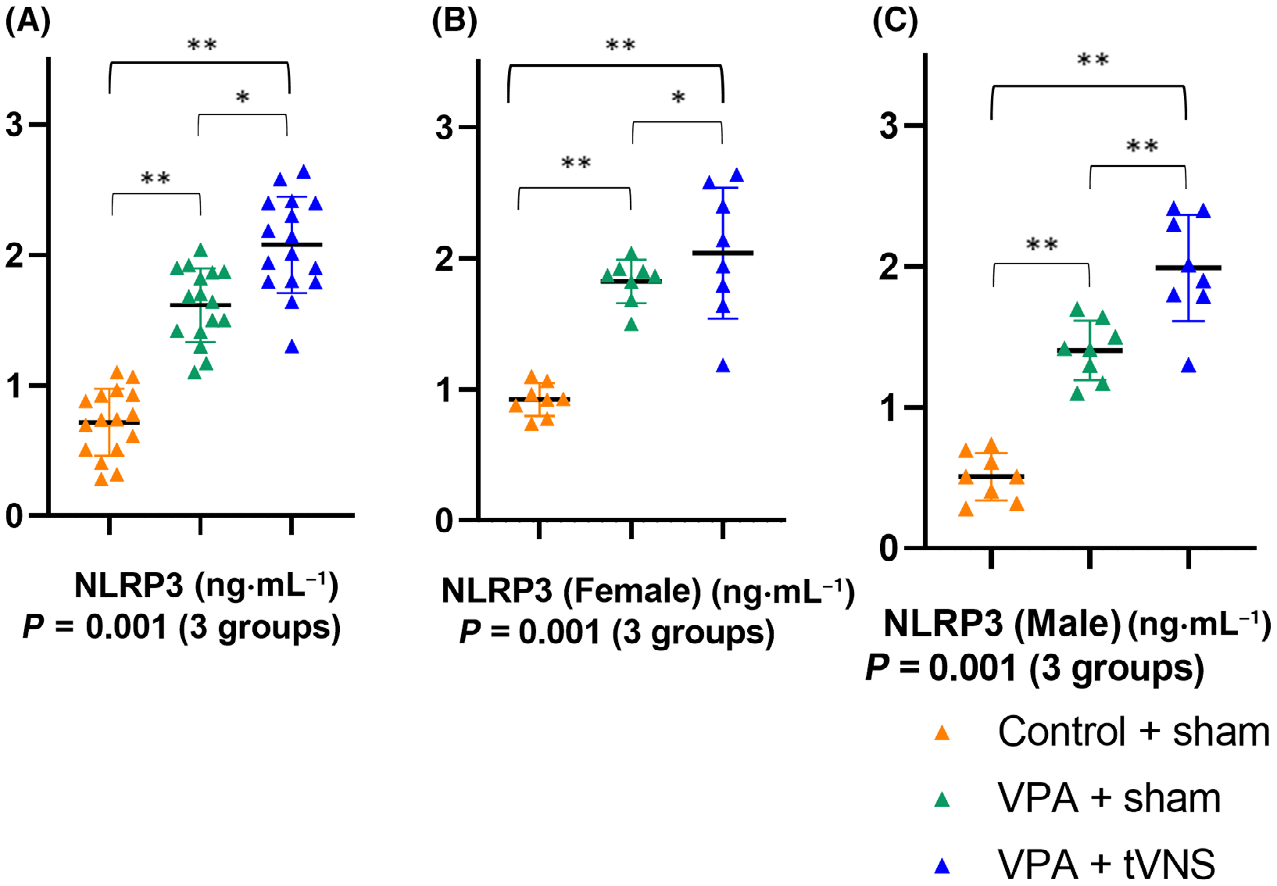
(A) Comparison of brain NLRP3 levels (*F* = 18.469, *P* = 0.001) between Control + sham (*n* = 16), VPA + sham (*n* = 16), VPA + tVNS (*n* = 16). Brain NLRP3 levels in both (B) female (*P* = 0.001) and (C) male (*P* = 0.001) mice were higher in the VPA + sham group compared to the Control + sham group. Statistical analysis was performed with One‐way ANOVA. *Post‐hoc* analyses were performed using the Bonferroni test. Data were shown as mean ± SD. *P‐value < 0.05, **P < 0.01.

Brain NLRP3 (*P* = 0.001) levels were significantly different across groups (*P* < 0.05). (Fig. 4).

Brain NLRP3 levels in both female (1.82 ± 0.16 vs. 0.92 ± 0.12, *P* = 0.001) and male (1.40 ± 0.21 vs. 0.50 ± 0.16, *P* = 0.001) mice were higher in the VPA + sham group compared to the Control + sham group. tVNS application increased brain NLRP3 levels in both female (2.16 ± 0.36 vs 1.82 ± 0.16, *P* = 0.03) and male (1.98 ± 0.37 vs 1.40 ± 0.21, *P* = 0.001) mice compared to VPA + sham.

**Fig. 5:**
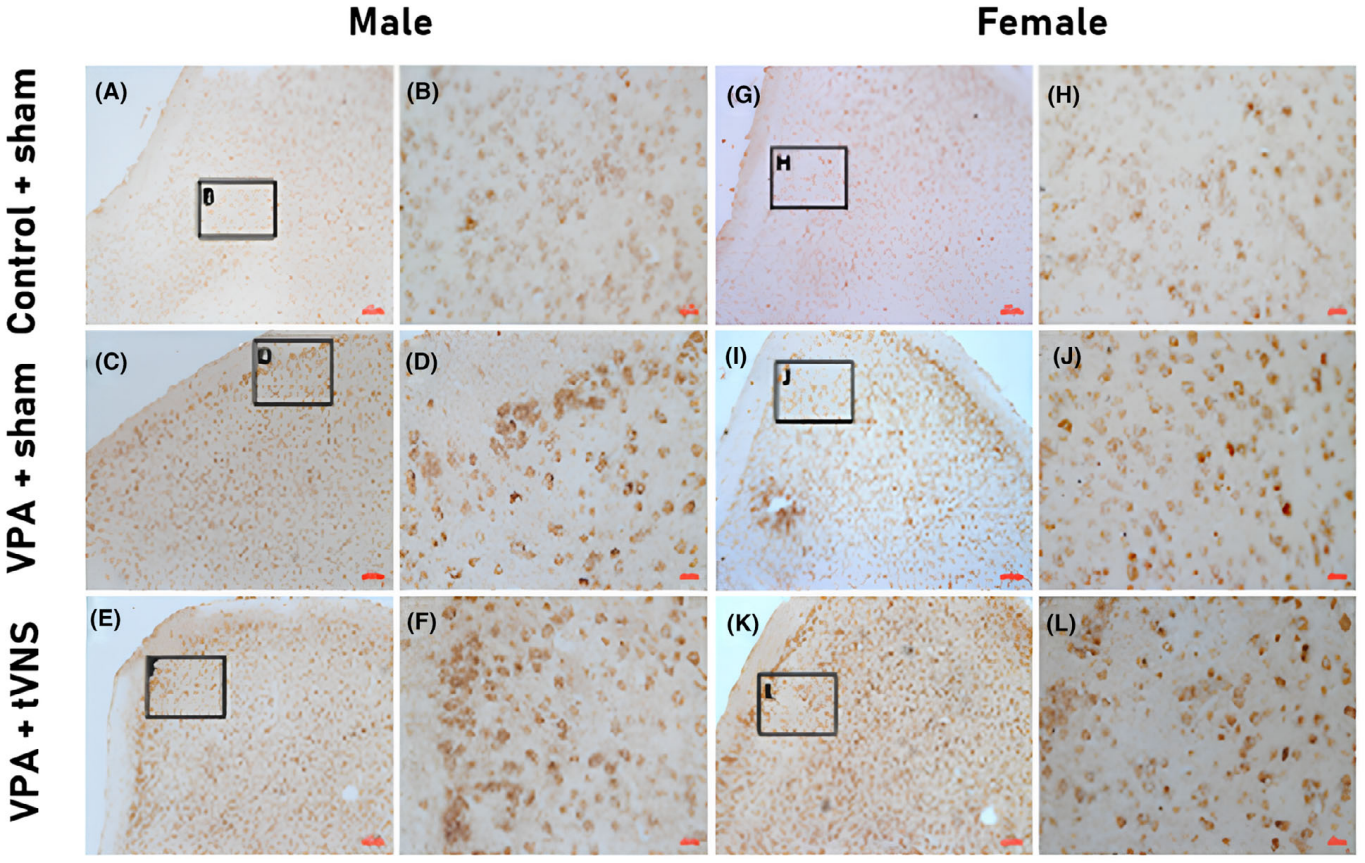
NLRP3 immunohistochemistry of prefrontal cortex anterior cingulate area in male (A–F) and female (G–L) mouse. A robust increase of cytoplasmic NLRP3 immunoreactivity is seen in the VPA + sham (C, D, I, J) and VPA + tVNS group (E, F, K, L) compared to the Control + sham group (A, B, G, H) (magnification 10× A, C, E, G, I, K and 40× B, D, F, H, J, L). Scale bars in images A, C, E, G, I, K = 100 μm. Scale bars in images B, D, F, H, J, L = 20 μm.

Representative immunohistochemistry (IHC) staining images of NLRP3 in the mouse brain cortex are shown at 100×, 400× magnification. NLRP3 expression increased in the VPA + sham and VPA + tVNS groups (Fig. 5).

## Discussion

Animal models are indispensable for advancing our understanding of the core biological mechanisms associated with ASD [23]. Therefore, in this study, we utilized C57BL/6 mice and established a model using VPA. This study proved that a single dose of VPA administered on day 12.5 of the embryonic period caused cytokine dysregulation and elevated inflammasome levels in adult mice. tVNS application increased brain NLRP3 levels and reduced serum IL‐1β and IL‐22 levels in the autism model.

The use of neuromodulation techniques in treating autism is increasing, with VNS being one of the prominent methods [57, 58, 59]. VNS therapy was first given to patients in 1988 and approved in 1994 as an adjunct medical treatment for patients with refractory epilepsy [60]. Increased levels of inflammatory cytokines and their imbalances within the serum and cerebrospinal fluid of individuals with autism may be associated with impaired cognition and behavior [45]. Experts view VNS as a potential treatment for autism, and attention is drawn to the effect of VNS on cytokines [26, 61, 62, 63, 64].

The vagus nerve has a critical role in homeostasis in the body, providing a natural defense against inflammation and essential roles in neuronal, endocrine, and behavioral responses [35, 65, 66].

Studies show vagal stimulation is anti‐inflammatory by reducing inflammatory markers [67]. In our study, serum IL‐1β and IL‐22 levels decreased significantly in the VPA + tVNS group compared to the VPA + sham group. Still, we observed an opposite effect in the brain, and NLRP3 levels increased significantly.

A systematic review and meta‐analysis by Masi *et al*. [68] on cytokine alterations in ASD demonstrated an increased inflammatory status and altered cytokine profile in autism.

In various studies, TNF‐α, IL‐1β, and IL‐6 levels were found to be higher in autism groups compared to controls [69, 70]. However, our study did not find a significant difference in TNF‐α levels in male and female mice in the VPA + sham group.

We demonstrated a difference in effect between genders on cytokines on IL‐1β levels in autism. Compared to the Control + sham, IL‐1β levels in the VPA + sham group decreased significantly in females and increased dramatically in males. This suggests that gender may need to be considered in biomarker research for autism.

Elevated IL‐6 levels are characteristic of individuals with autism [71, 72]. We observed a significant IL‐6 increase in the VPA + sham group compared to controls only in male mice. The difference in females was not statistically significant.

Dysregulated cytokine chemokine profiles have been reported in autism [73, 74, 75]. The IL‐10 family cytokines play crucial roles in immune regulation by suppressing excessive inflammatory responses and supporting tissue repair mechanisms. IL 22, a member of the IL‐10 family is produced by both innate and adaptive immune cells [76, 77]. In our study, we observed significant differences in serum IL‐22 levels between groups. The implications of chronic neurological inflammation and immune dysregulation in autism warrant further investigation [78].

Inflammasomes are multimeric proteins whose activity is involved in regulating inflammatory responses, resulting in the production of proinflammatory cytokines [79]. Research has demonstrated the activation of multiple inflammatory complexes in autism, and one of them is NLRP3 [80, 81]. NLRP3 inflammasome is a cytosolic receptor protein primarily found in immune and inflammatory cells after activation by inflammatory stimuli [79, 81, 82, 83].

NLRP3 inflammasome activation is predicted to have an essential place in the development of autism. ST36 acupuncture inhibited NLRP3 inflammasome activation and alleviated prefrontal cortex‐related behavioral impairment in a study utilizing VPA‐induced rat models of autism [84]. Szabo *et al*. [85] demonstrated in their study using a maternal immune activation (MIA) mouse model of autism (C57BL/6) that maternal treatment with an NLRP3 antagonist (blocker) and a neutralizing IL‐1β antibody during pregnancy prevented the development of autistic traits in male offspring mice. Furthermore, children with ASD have shown increased NLRP3 inflammasome activity compared to their siblings and healthy controls, suggesting that inflammasome activity may play a role in neuroinflammation associated with ASD [81].

We found a significant difference between groups regarding NLRP3 levels (*P* < 0.05). In our study, in both male and female mice, NLRP3 levels in the VPA + sham group compared to Control + sham were significantly higher. Additionally, tVNS application significantly increased NLRP3 levels in both genders compared to the VPA + sham group. The neuroprotective effects of VNS in early brain damage after traumatic brain injury were examined by Tang *et al*. [86] and it was found that VNS reduced NLRP3 levels, but its chronic effects were not investigated. In our study, we created an adult animal model and interestingly determined that tVNS application increased brain NLRP3 levels. We think new research is needed in young and adult autism models. Due to serum's dynamic nature as a fluid, we consider it important to take into account differences between serum and brain tissue in these studies. The social deficit is one of the symptoms of autism where networks between major cortical, subcortical structures, and neuromodulatory systems mediate social behavior [87, 88, 89]. Therefore, in this study, brain NLRP3 levels in the anterior cingulate cortex, one of the primary cortical areas, are shown as representative. The ELISA and IHC methods show parallelism in their results, indicating consistency between the two of them.

It is increasingly recognized that inflammation significantly contributes to central nervous system (CNS) damage in developing and adult brains. In several studies, inflammatory mediators affect the brain during development, with ASD associated with inflammation in early life [90, 91, 92].

Our study investigated the effects of noninvasive trans auricular vagus nerve stimulation on the inflammatory response in one model of autism for the first time. Our findings provided evidence for cytokine dysregulation in autism model and the role of sexual dimorphism in inflammatory response, suggesting a potential role of cytokine monitoring. One of the limitations of this study is that regional brain analyses were not performed. Also, behavioral experiments and neurotransmitter studies needed to be improved. Although the VPA‐induced model is essential to understanding the mechanisms of autism, it cannot fully reflect the condition in human beings due to the species differences.

## Conclusion

In our study, the cytokine profile was altered in the VPA‐induced adult autism mouse model. Furthermore, tVNS application altered the inflammatory response and resulted in increased brain NLPR3 levels in both sexes. However, additional studies are required to determine whether this inflammatory response might be beneficial or detrimental in autism. Inflammation is essential in both the etiology and as potential biomarkers of autism. Stimulation of the vagus nerve, which plays a crucial role in the communication between the brain and the intestines, is considered a potential therapeutic target in many neuropsychiatric disorders, including autism, although many uncertainties remain. Although our data shed light on the inflammatory perspective on the etiopathogenesis of autism, essential points, such as the beneficial role of the inflammatory response, still need to be further clarified. We showed that gender and individual differences in autism should always be considered, and that cytokine monitoring could be helpful. However, additional studies are required to better understand this neurodevelopmental disorder, which incidence is increasing rapidly, and to find biomarkers and develop treatment methods.

## Conflict of interest

The authors declare no conflict of interest.

### Peer review

The peer review history for this article is available at https://www.webofscience.com/api/gateway/wos/peer‐review/10.1002/2211‐5463.13889.

## Author contributions

NB, MB, and HB designed the study. HGD, AÇ, EA, SÖD and ET collected and analyzed the data. HGD, NB, MB, AÇ, EA, SÖD, ET and HB drafted the manuscript. All authors reviewed and approved the final manuscript. This original article was produced from the doctoral thesis of the first author.

## Supporting information


**Video S1.** Inflammatory response in autism and effect of tVNS

## Data Availability

The data supporting the findings of this study are available from Hale Gök Dağıdır (hgokdagidir@gmail.com) if requested.

## References

[1] Sanchack KE and Thomas CA (2016) Autism Spectrum disorder: primary care principles. Am Fam Physician 94, 972–979. 28075089

[2] Chiarotti F and Venerosi A (2020) Epidemiology of autism Spectrum disorders: a review of worldwide prevalence estimates since 2014. Brain Sci 10, 274. 32370097 10.3390/brainsci10050274 PMC7288022

[3] Zeidan J , Fombonne E , Scorah J , Ibrahim A , Durkin MS , Saxena S , Yusuf A , Shih A and Elsabbagh M (2022) Global prevalence of autism: a systematic review update. Autism Res 15, 778–790. 35238171 10.1002/aur.2696 PMC9310578

[4] Wang J , Ma B , Wang J , Zhang Z and Chen O (2022) Global prevalence of autism spectrum disorder and its gastrointestinal symptoms: a systematic review and meta‐analysis. Front Psych 13, 963102. 10.3389/fpsyt.2022.963102 PMC9445193 36081466

[5] Lai MC , Lombardo MV and Baron‐Cohen S (2014) Autism. Lancet 383, 896–910. 24074734 10.1016/S0140-6736(13)61539-1

[6] Battle DE (2013) Diagnostic and statistical manual of mental disorders (DSM). Codas 25, 191–192. 24413388 10.1590/s2317-17822013000200017

[7] Canitano R (2007) Epilepsy in autism spectrum disorders. Eur Child Adolesc Psychiatry 16, 61–66. 16932856 10.1007/s00787-006-0563-2

[8] Tuchman R and Rapin I (2002) Epilepsy in autism. Lancet Neurol 1, 352–358. 12849396 10.1016/s1474-4422(02)00160-6

[9] Chaste P and Leboyer M (2012) Autism risk factors: genes, environment, and gene‐environment interactions. Dialogues Clin Neurosci 14, 281–292. 23226953 10.31887/DCNS.2012.14.3/pchaste PMC3513682

[10] Gevi F , Zolla L , Gabriele S and Persico AM (2016) Urinary metabolomics of young Italian autistic children supports abnormal tryptophan and purine metabolism. Mol Autism 7, 47. 27904735 10.1186/s13229-016-0109-5 PMC5121959

[11] Volkmar FR and Pauls D (2003) Autism. Lancet 362, 1133–1141. 14550703 10.1016/S0140-6736(03)14471-6

[12] Howsmon DP , Kruger U , Melnyk S , James SJ and Hahn J (2017) Classification and adaptive behavior prediction of children with autism spectrum disorder based upon multivariate data analysis of markers of oxidative stress and DNA methylation. PLoS Comput Biol 13, e1005385. 28301476 10.1371/journal.pcbi.1005385 PMC5354243

[13] Ratajczak HV (2011) Theoretical aspects of autism: causes—a review. J Immunotoxicol 8, 68–79. 21299355 10.3109/1547691X.2010.545086

[14] Mukherjee SB (2017) Autism Spectrum disorders – diagnosis and management. Indian J Pediatr 84, 307–314. 28101829 10.1007/s12098-016-2272-2

[15] Park G , Jang WE , Kim S , Gonzales EL , Ji J , Choi S , Kim Y , Park JH , Mohammad HB , Bang G *et al*. (2023) Dysregulation of the Wnt/beta‐catenin signaling pathway via Rnf146 upregulation in a VPA‐induced mouse model of autism spectrum disorder. Exp Mol Med 55, 1783–1794. 37524878 10.1038/s12276-023-01065-2 PMC10474298

[16] Ergaz Z , Weinstein‐Fudim L and Ornoy A (2016) Genetic and non‐genetic animal models for autism spectrum disorders (ASD). Reprod Toxicol 64, 116–140. 27142188 10.1016/j.reprotox.2016.04.024

[17] Favre MR , Barkat TR , Lamendola D , Khazen G , Markram H and Markram K (2013) General developmental health in the VPA‐rat model of autism. Front Behav Neurosci 7, 88. 23898245 10.3389/fnbeh.2013.00088 PMC3721005

[18] Nicolini C and Fahnestock M (2018) The valproic acid‐induced rodent model of autism. Exp Neurol 299, 217–227. 28472621 10.1016/j.expneurol.2017.04.017

[19] Chaliha D , Albrecht M , Vaccarezza M , Takechi R , Lam V , Al‐Salami H and Mamo J (2020) A systematic review of the valproic‐acid‐induced rodent model of autism. Dev Neurosci 42, 12–48. 32810856 10.1159/000509109

[20] Halladay AK , Amaral D , Aschner M , Bolivar VJ , Bowman A , DiCicco‐Bloom E , Hyman SL , Keller F , Lein P , Pessah I *et al*. (2009) Animal models of autism spectrum disorders: information for neurotoxicologists. Neurotoxicology 30, 811–821. 19596370 10.1016/j.neuro.2009.07.002 PMC3014989

[21] Lazaro MT and Golshani P (2015) The utility of rodent models of autism spectrum disorders. Curr Opin Neurol 28, 103–109. 25734952 10.1097/WCO.0000000000000183 PMC4476903

[22] Schwartzer JJ , Koenig CM and Berman RF (2013) Using mouse models of autism spectrum disorders to study the neurotoxicology of gene‐environment interactions. Neurotoxicol Teratol 36, 17–35. 23010509 10.1016/j.ntt.2012.08.007 PMC3538113

[23] Jiao D , Xu Y , Tian F , Zhou Y , Chen D and Wang Y (2024) Establishment of animal models and behavioral studies for autism spectrum disorders. J Int Med Res 52, 3000605241245293. 38619175 10.1177/03000605241245293 PMC11022675

[24] Derakhshan N (2015) Vagal nerve stimulation for the treatment of autism. Ment Illn 7, 5788. 26266025 10.4081/mi.2015.5788 PMC4508631

[25] Porges SW (2003) The polyvagal theory: phylogenetic contributions to social behavior. Physiol Behav 79, 503–513. 12954445 10.1016/s0031-9384(03)00156-2

[26] van Hoorn A , Carpenter T , Oak K , Laugharne R , Ring H and Shankar R (2019) Neuromodulation of autism spectrum disorders using vagal nerve stimulation. J Clin Neurosci 63, 8–12. 30732986 10.1016/j.jocn.2019.01.042

[27] Klusek J , Martin GE and Losh M (2013) Physiological arousal in autism and fragile X syndrome: group comparisons and links with pragmatic language. Am J Intellect Dev Disabil 118, 475–495. 24432860 10.1352/1944.7558-118.6.475 PMC3928802

[28] Porges SW (2007) The polyvagal perspective. Biol Psychol 74, 116–143. 17049418 10.1016/j.biopsycho.2006.06.009 PMC1868418

[29] Condy EE , Scarpa A and Friedman BH (2019) Restricted repetitive behaviors in autism spectrum disorder: a systematic review from the neurovisceral integration perspective. Biol Psychol 148, 107739. 31415791 10.1016/j.biopsycho.2019.107739

[30] Cohen S , Masyn K , Mastergeorge A and Hessl D (2015) Psychophysiological responses to emotional stimuli in children and adolescents with autism and fragile X syndrome. J Clin Child Adolesc Psychol 44, 250–263. 24156344 10.1080/15374416.2013.843462 PMC3999342

[31] Beopoulos A , Gea M , Fasano A and Iris F (2021) Autonomic nervous system neuroanatomical alterations could provoke and maintain gastrointestinal dysbiosis in autism Spectrum disorder (ASD): a novel microbiome‐host interaction mechanistic hypothesis. Nutrients 14, 65. 35010940 10.3390/nu14010065 PMC8746684

[32] Ming X , Patel R , Kang V , Chokroverty S and Julu PO (2016) Respiratory and autonomic dysfunction in children with autism spectrum disorders. Brain Dev 38, 225–232. 26235973 10.1016/j.braindev.2015.07.003

[33] Ming X , Julu PO , Brimacombe M , Connor S and Daniels ML (2005) Reduced cardiac parasympathetic activity in children with autism. Brain Dev 27, 509–516. 16198209 10.1016/j.braindev.2005.01.003

[34] Strigo IA and Craig AD (2016) Interoception, homeostatic emotions and sympathovagal balance. Philos Trans R Soc Lond B Biol Sci 371, 20160010. 28080968 10.1098/rstb.2016.0010 PMC5062099

[35] Engineer CT , Hays SA and Kilgard MP (2017) Vagus nerve stimulation as a potential adjuvant to behavioral therapy for autism and other neurodevelopmental disorders. J Neurodev Disord 9, 20. 28690686 10.1186/s11689-017-9203-z PMC5496407

[36] Klusek J , Fairchild AJ and Roberts JE (2019) Vagal tone as a putative mechanism for pragmatic competence: an investigation of carriers of the FMR1 premutation. J Autism Dev Disord 49, 197–208. 30097759 10.1007/s10803-018-3714-7 PMC6855249

[37] Roberts JE , Tonnsen B , Robinson A and Shinkareva SV (2012) Heart activity and autistic behavior in infants and toddlers with fragile X syndrome. Am J Intellect Dev Disabil 117, 90–102. 22515825 10.1352/1944-7558-117.2.90 PMC3987776

[38] Hull MM , Madhavan D and Zaroff CM (2015) Autistic spectrum disorder, epilepsy, and vagus nerve stimulation. Childs Nerv Syst 31, 1377–1385. 25922052 10.1007/s00381-015-2720-8

[39] Shivaswamy T , Souza RR , Engineer CT and McIntyre CK (2022) Vagus nerve stimulation as a treatment for fear and anxiety in individuals with autism Spectrum disorder. J Psychiatr Brain Sci 7, e220007. 36303861 10.20900/jpbs.20220007 PMC9600938

[40] Black B , Hunter S , Cottrell H , Dar R , Takahashi N , Ferguson BJ , Valter Y , Porges E , Datta A and Beversdorf DQ (2023) Remotely supervised at‐home delivery of taVNS for autism spectrum disorder: feasibility and initial efficacy. Front Psych 14, 1238328. 10.3389/fpsyt.2023.1238328 PMC10568329 37840787

[41] Park YD (2003) The effects of vagus nerve stimulation therapy on patients with intractable seizures and either Landau‐Kleffner syndrome or autism. Epilepsy Behav 4, 286–290. 12791330 10.1016/s1525-5050(03)00080-5

[42] Huffman WJ , Subramaniyan S , Rodriguiz RM , Wetsel WC , Grill WM and Terrando N (2019) Modulation of neuroinflammation and memory dysfunction using percutaneous vagus nerve stimulation in mice. Brain Stimul 12, 19–29. 30337243 10.1016/j.brs.2018.10.005 PMC6301148

[43] Xu N , Li X and Zhong Y (2015) Inflammatory cytokines: potential biomarkers of immunologic dysfunction in autism spectrum disorders. Mediators Inflamm 2015, 531518. 25729218 10.1155/2015/531518 PMC4333561

[44] Madore C , Leyrolle Q , Lacabanne C , Benmamar‐Badel A , Joffre C , Nadjar A and Layé S (2016) Neuroinflammation in autism: plausible role of maternal inflammation, dietary omega 3, and microbiota. Neural Plast 2016, 3597209. 27840741 10.1155/2016/3597209 PMC5093279

[45] Ashwood P , Krakowiak P , Hertz‐Picciotto I , Hansen R , Pessah I and Van de Water J (2011) Elevated plasma cytokines in autism spectrum disorders provide evidence of immune dysfunction and are associated with impaired behavioral outcome. Brain Behav Immun 25, 40–45. 20705131 10.1016/j.bbi.2010.08.003 PMC2991432

[46] Onore C , Careaga M and Ashwood P (2012) The role of immune dysfunction in the pathophysiology of autism. Brain Behav Immun 26, 383–392. 21906670 10.1016/j.bbi.2011.08.007 PMC3418145

[47] Meltzer A and Van de Water J (2017) The role of the immune system in autism Spectrum disorder. Neuropsychopharmacology 42, 284–298. 27534269 10.1038/npp.2016.158 PMC5143489

[48] Bjorklund G , Meguid NA , El‐Ansary A , El‐Bana MA , Dadar M , Aaseth J , Hemimi M , Osredkar J and Chirumbolo S (2018) Diagnostic and severity‐tracking biomarkers for autism Spectrum disorder. J Mol Neurosci 66, 492–511. 30357679 10.1007/s12031-018-1192-1

[49] Zerbo O , Yoshida C , Grether JK , Van de Water J , Ashwood P , Delorenze GN , Hansen RL , Kharrazi M and Croen LA (2014) Neonatal cytokines and chemokines and risk of autism Spectrum disorder: the early markers for autism (EMA) study: a case‐control study. J Neuroinflammation 11, 113. 24951035 10.1186/1742-2094-11-113 PMC4080514

[50] Ravaccia D and Ghafourian T (2020) Critical role of the maternal immune system in the pathogenesis of autism Spectrum disorder. Biomedicine 8, 557. 10.3390/biomedicines8120557 PMC7760377 33271759

[51] Hays SA , Rennaker RL 2nd and Kilgard MP (2023) How to fail with paired VNS therapy. Brain Stimul 16, 1252–1258. 37595833 10.1016/j.brs.2023.08.009 PMC11650123

[52] Kumaria A and Sitaraman M (2019) Can vagus nerve stimulation improve social cognition in autism? Cortex 115, 350–351. 29615199 10.1016/j.cortex.2018.02.020

[53] Ding JJ , Liu P , Rebernig H , Suller‐Marti A , Parrent AG , Burneo JG , Hammond RR , Ang L‐C and Zhang Q (2021) Vagus nerve stimulation does not alter brainstem nuclei morphology in patients with refractory epilepsy. Epilepsy Behav 118, 107940. 33838622 10.1016/j.yebeh.2021.107940

[54] Robinson‐Agramonte MLA , Noris Garcia E , Fraga Guerra J , Vega Hurtado Y , Antonucci N , Semprun‐Hernandez N , Schultz S and Siniscalco D (2022) Immune dysregulation in autism Spectrum disorder: what do we know about it? Int J Mol Sci 23, 3033. 35328471 10.3390/ijms23063033 PMC8955336

[55] Zhang L , Bang S , He Q , Matsuda M , Luo X , Jiang YH and Ji R‐R (2023) SHANK3 in vagal sensory neurons regulates body temperature, systemic inflammation, and sepsis. Front Immunol 14, 1124356. 36845137 10.3389/fimmu.2023.1124356 PMC9944123

[56] Tomaiuolo P , Piras IS , Sain SB , Picinelli C , Baccarin M , Castronovo P , Morelli MJ , Lazarevic D , Scattoni ML , Tonon G *et al*. (2023) RNA sequencing of blood from sex‐ and age‐matched discordant siblings supports immune and transcriptional dysregulation in autism spectrum disorder. Sci Rep 13, 807. 36646776 10.1038/s41598-023-27378-w PMC9842630

[57] Xiao L , Huo X , Wang Y , Li W , Li M , Wang C , Wang F and Sun T (2023) A bibliometric analysis of global research status and trends in neuromodulation techniques in the treatment of autism spectrum disorder. BMC Psychiatry 23, 183. 36941549 10.1186/s12888-023-04666-3 PMC10026211

[58] Barahona‐Correa JB , Velosa A , Chainho A , Lopes R and Oliveira‐Maia AJ (2018) Repetitive transcranial magnetic stimulation for treatment of autism Spectrum disorder: a systematic review and meta‐analysis. Front Integr Neurosci 12, 27. 30038561 10.3389/fnint.2018.00027 PMC6046620

[59] Warwick TC , Griffith J , Reyes B , Legesse B and Evans M (2007) Effects of vagus nerve stimulation in a patient with temporal lobe epilepsy and Asperger syndrome: case report and review of the literature. Epilepsy Behav 10, 344–347. 17300990 10.1016/j.yebeh.2007.01.001

[60] Vonck K , Raedt R , Naulaerts J , De Vogelaere F , Thiery E , Van Roost D , Aldenkamp B , Miatton M and Boon P (2014) Vagus nerve stimulation…25 years later! What do we know about the effects on cognition? Neurosci Biobehav Rev 45, 63–71. 24858008 10.1016/j.neubiorev.2014.05.005

[61] Wang Z , Yuan X , Zhang Q , Wen J , Cheng T , Qin X , Ji T , Shu X , Jiang Y , Liao J *et al*. (2022) Effects of stable Vagus nerve stimulation efficacy on autistic behaviors in ten pediatric patients with drug resistant epilepsy: an observational study. Front Pediatr 10, 846301. 35311037 10.3389/fped.2022.846301 PMC8924444

[62] Zimmerman AW , Jyonouchi H , Comi AM , Connors SL , Milstien S , Varsou A and Heyes MP (2005) Cerebrospinal fluid and serum markers of inflammation in autism. Pediatr Neurol 33, 195–201. 16139734 10.1016/j.pediatrneurol.2005.03.014

[63] Kelly JR , Minuto C , Cryan JF , Clarke G and Dinan TG (2017) Cross talk: the microbiota and neurodevelopmental disorders. Front Neurosci 11, 490. 28966571 10.3389/fnins.2017.00490 PMC5605633

[64] Sun K , Li Y , Zhai ZH , Yin HQ , Liang SL , Zhai F , Cui Y and Zhang G (2024) Effects of transcutaneous auricular vagus nerve stimulation and exploration of brain network mechanisms in children with high‐functioning autism spectrum disorder: study protocol for a randomized controlled trial. Front Psych 15, 1337101. 10.3389/fpsyt.2024.1337101 PMC10875019 38374975

[65] Roosevelt RW , Smith DC , Clough RW , Jensen RA and Browning RA (2006) Increased extracellular concentrations of norepinephrine in cortex and hippocampus following vagus nerve stimulation in the rat. Brain Res 1119, 124–132. 16962076 10.1016/j.brainres.2006.08.04 PMC1751174

[66] Ruffoli R , Giorgi FS , Pizzanelli C , Murri L , Paparelli A and Fornai F (2011) The chemical neuroanatomy of vagus nerve stimulation. J Chem Neuroanat 42, 288–296. 21167932 10.1016/j.jchemneu.2010.12.002

[67] Meregnani J , Clarencon D , Vivier M , Peinnequin A , Mouret C , Sinniger V , Picq C , Job A , Canini F , Jacquier‐Sarlin M *et al*. (2011) Anti‐inflammatory effect of vagus nerve stimulation in a rat model of inflammatory bowel disease. Auton Neurosci 160, 82–89. 21071287 10.1016/j.autneu.2010.10.007

[68] Masi A , Quintana DS , Glozier N , Lloyd AR , Hickie IB and Guastella AJ (2015) Cytokine aberrations in autism spectrum disorder: a systematic review and meta‐analysis. Mol Psychiatry 20, 440–446. 24934179 10.1038/mp.2014.59

[69] Jyonouchi H , Sun S and Le H (2001) Proinflammatory and regulatory cytokine production associated with innate and adaptive immune responses in children with autism spectrum disorders and developmental regression. J Neuroimmunol 120, 170–179. 11694332 10.1016/s0165-5728(01)00421-0

[70] Kutuk MO , Tufan E , Gokcen C , Kilicaslan F , Karadag M , Mutluer T , Yektas C , Coban N , Kandemir H , Buber A *et al*. (2020) Cytokine expression profiles in autism spectrum disorder: a multi‐center study from Turkey. Cytokine 133, 155152. 32563959 10.1016/j.cyto.2020.155152

[71] Wei H , Alberts I and Li X (2013) Brain IL‐6 and autism. Neuroscience 252, 320–325. 23994594 10.1016/j.neuroscience.2013.08.025

[72] Li X , Chauhan A , Sheikh AM , Patil S , Chauhan V , Li XM , Ji L , Brown T and Malik M (2009) Elevated immune response in the brain of autistic patients. J Neuroimmunol 207, 111–116. 19157572 10.1016/j.jneuroim.2008.12.002 PMC2770268

[73] Pecorelli A , Cervellati F , Belmonte G , Montagner G , Waldon P , Hayek J , Gambari R and Valacchi G (2016) Cytokines profile and peripheral blood mononuclear cells morphology in Rett and autistic patients. Cytokine 77, 180–188. 26471937 10.1016/j.cyto.2015.10.002

[74] Runge K , Fiebich BL , Kuzior H , Rausch J , Maier SJ , Dersch R , Nickel K , Domschke K , van Elst LT and Endres D (2023) Altered cytokine levels in the cerebrospinal fluid of adult patients with autism spectrum disorder. J Psychiatr Res 158, 134–142. 36584491 10.1016/j.jpsychires.2022.12.032

[75] Jyonouchi H and Geng L (2019) Associations between monocyte and T cell cytokine profiles in autism Spectrum disorders: effects of dysregulated innate immune responses on adaptive responses to recall antigens in a subset of ASD children. Int J Mol Sci 20, 4731. 31554204 10.3390/ijms20194731 PMC6801811

[76] Ouyang W and O'Garra A (2019) IL‐10 family cytokines IL‐10 and IL‐22: from basic science to clinical translation. Immunity 50, 871–891. 30995504 10.1016/j.immuni.2019.03.020

[77] Dudakov JA , Hanash AM and van den Brink MR (2015) Interleukin‐22: immunobiology and pathology. Annu Rev Immunol 33, 747–785. 25706098 10.1146/annurev-immunol-032414-112123 PMC4407497

[78] Moaaz M , Youssry S , Elfatatry A and El Rahman MA (2019) Th17/Treg cells imbalance and their related cytokines (IL‐17, IL‐10 and TGF‐beta) in children with autism spectrum disorder. J Neuroimmunol 337, 577071. 31671361 10.1016/j.jneuroim.2019.577071

[79] Schroder K and Tschopp J (2010) The inflammasomes. Cell 140, 821–832. 20303873 10.1016/j.cell.2010.01.040

[80] Frasch MG , Yoon BJ , Helbing DL , Snir G , Antonelli MC and Bauer R (2023) Autism Spectrum disorder: a neuro‐Immunometabolic hypothesis of the developmental origins. Biology (Basel) 12, 914. 37508346 10.3390/biology12070914 PMC10375982

[81] Saresella M , Piancone F , Marventano I , Zoppis M , Hernis A , Zanette M , Trabattoni D , Chiappedi M , Ghezzo A , Canevini MP *et al*. (2016) Multiple inflammasome complexes are activated in autistic spectrum disorders. Brain Behav Immun 57, 125–133. 26979869 10.1016/j.bbi.2016.03.009

[82] Shao BZ , Xu ZQ , Han BZ , Su DF and Liu C (2015) NLRP3 inflammasome and its inhibitors: a review. Front Pharmacol 6, 262. 26594174 10.3389/fphar.2015.00262 PMC4633676

[83] Shen Y , Qian L , Luo H , Li X , Ruan Y , Fan R , Si Z , Chen Y , Li L and Liu Y (2022) The significance of NLRP inflammasome in neuropsychiatric disorders. Brain Sci 12, 1057. 36009120 10.3390/brainsci12081057 PMC9406040

[84] Zhao P , Fu H , Cheng H , Zheng R , Yuan D , Yang J , Li S , Li E and Li L (2022) Acupuncture at ST36 alleviates the behavioral disorder of autistic rats by inhibiting TXNIP‐mediated activation of NLRP3. J Neuropathol Exp Neurol 81, 127–134. 35015875 10.1093/jnen/nlab132

[85] Szabo D , Tod P , Goloncser F , Roman V , Lendvai B , Otrokocsi L and Sperlágh B (2022) Maternal P2X7 receptor inhibition prevents autism‐like phenotype in male mouse offspring through the NLRP3‐IL‐1beta pathway. Brain Behav Immun 101, 318–332. 35065198 10.1016/j.bbi.2022.01.015

[86] Tang Y , Dong X , Chen G , Ye W , Kang J , Tang Y and Feng Z (2020) Vagus nerve stimulation attenuates early traumatic brain injury by regulating the NF‐kappaB/NLRP3 signaling pathway. Neurorehabil Neural Repair 34, 831–843. 32772884 10.1177/1545968320948065

[87] Sato M , Nakai N , Fujima S , Choe KY and Takumi T (2023) Social circuits and their dysfunction in autism spectrum disorder. Mol Psychiatry 28, 3194–3206. 37612363 10.1038/s41380-023-02201-0 PMC10618103

[88] Simms ML , Kemper TL , Timbie CM , Bauman ML and Blatt GJ (2009) The anterior cingulate cortex in autism: heterogeneity of qualitative and quantitative cytoarchitectonic features suggests possible subgroups. Acta Neuropathol 118, 673–684. 19590881 10.1007/s00401-009-0568-2

[89] Guo B , Chen J , Chen Q , Ren K , Feng D , Mao H , Yao H , Yang J , Liu H , Liu Y *et al*. (2019) Anterior cingulate cortex dysfunction underlies social deficits in Shank3 mutant mice. Nat Neurosci 22, 1223–1234. 31332372 10.1038/s41593-019-0445-9

[90] Jiang CC , Lin LS , Long S , Ke XY , Fukunaga K , Lu YM and Han F (2022) Signalling pathways in autism spectrum disorder: mechanisms and therapeutic implications. Signal Transduct Target Ther 7, 229. 35817793 10.1038/s41392-022-01081-0 PMC9273593

[91] Faust TE , Gunner G and Schafer DP (2021) Mechanisms governing activity‐dependent synaptic pruning in the developing mammalian CNS. Nat Rev Neurosci 22, 657–673. 34545240 10.1038/s41583-021-00507-y PMC8541743

[92] Theoharides TC , Tsilioni I , Patel AB and Doyle R (2016) Atopic diseases and inflammation of the brain in the pathogenesis of autism spectrum disorders. Transl Psychiatry 6, e844. 27351598 10.1038/tp.2016.77 PMC4931610

