# Supplementary material for: tVNS alters inflammatory response in adult VPA‐induced mouse model of autism: evidence for sexual dimorphism
Source: FEBS Open Bio. 2024 Oct 14;15(1):69–80. doi: 10.1002/2211-5463.13889 (PMC11705413; doi:10.1002/2211-5463.13889)
Supplement: Supplementary file 1 — Video S1. Inflammatory response in autism and effect of tVNS [file FEB4-15-69-s001.zip › VİDEO TEXT.docx]

Hello

My name is Hale Gök Dağıdır

I am a postdoctoral researcher at Gazi University Neuroscience and Neurotechnology Center of Excellence. I am very pleased to tell you about our work with you.

In this study, we aimed to examine the effect of bilateral transcutaneous auricular vagus nerve stimulation (tVNS) on inflammatory response in an adult valproic acid(VPA) induced mouse model of autism.

The study included three groups: VPA + tVNS, VPA + Sham and Control +Sham. Each group included 8 male and 8 female mice.

Serum interleukin 1 Beta (IL1B), IL6, IL22 and brain NLRP3 levels were significantly different across groups.

We conclude that cytokine dysregulation is associated with the VPA induced adult autism model and the Inflammatory response is more pronounced in male mice. tVNS application altered the inflammatory response and increased brain NLRP3 levels in both sexes.

If you would like to receive more information about our work, please do not hesitate to reach out to our team. We sincerely thank you.
