# Supplementary figures and images for: tVNS alters inflammatory response in adult VPA‐induced mouse model of autism: evidence for sexual dimorphism
Source: FEBS Open Bio. 2024 Oct 14;15(1):69–80. doi: 10.1002/2211-5463.13889 (PMC11705413; doi:10.1002/2211-5463.13889)

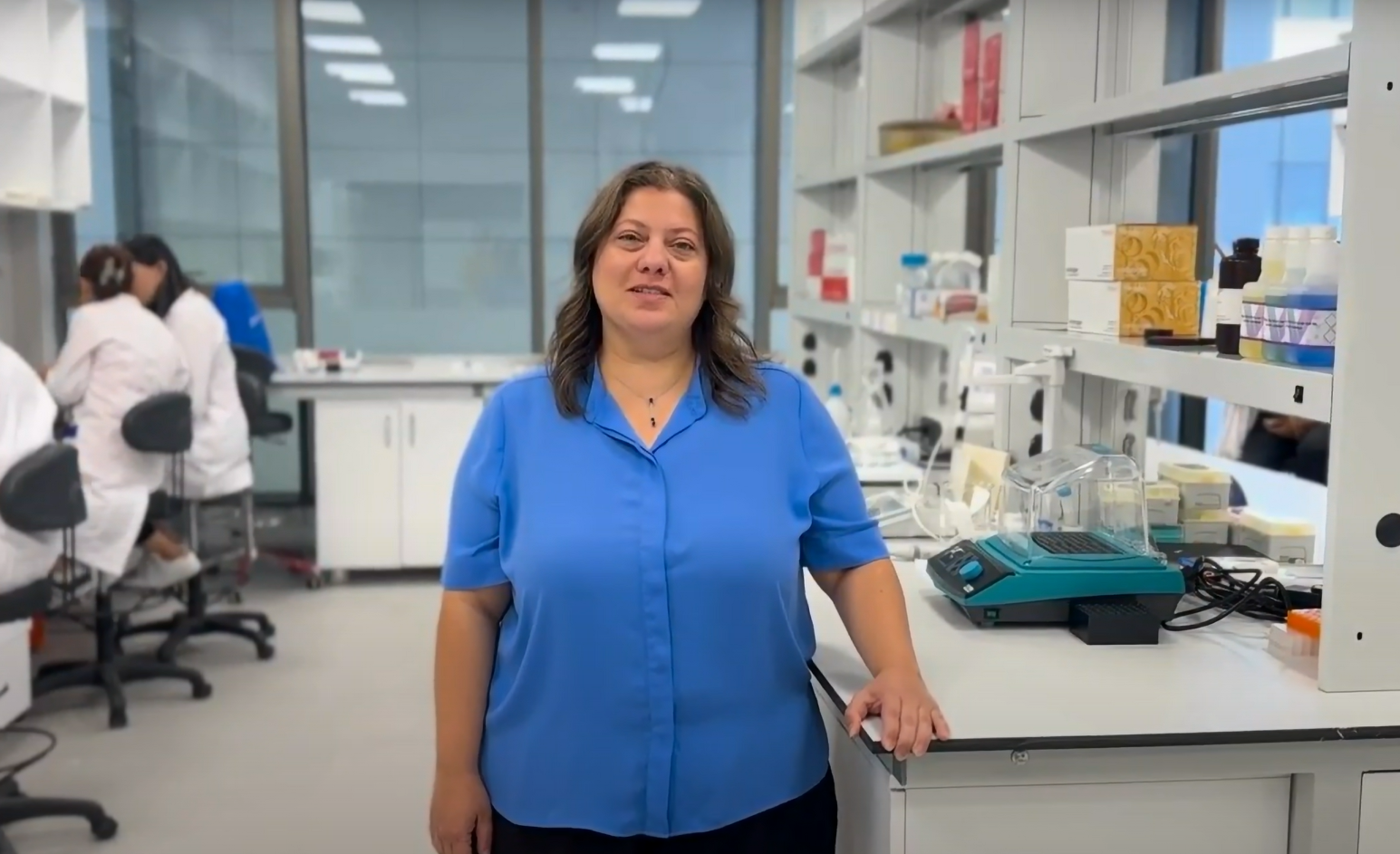

Supplement: Supplementary file 1 — Video S1. Inflammatory response in autism and effect of tVNS [file FEB4-15-69-s001.zip › FEBSOPEN-24-0067.R2_Thumbnail for video abstract.png]
